# Identification of Potential Core Genes for the Rupture of Intracranial Aneurysms by a Bioinformatics Analysis

**DOI:** 10.3389/fgene.2022.875007

**Published:** 2022-03-30

**Authors:** Yuan Lin, Hai-Ying Ma, Yi Wang, Jiang He, Heng-Jian Liu

**Affiliations:** Changzhou Hospital of Traditional Chinese Medicine, Changzhou, China

**Keywords:** bioinformatics analysis, rupture, unruptured aneurysm, differentially expressed genes, gene expression omnibus

## Abstract

**Background:** Previous studies, using autopsy and angiography, have shown that 3.6–6% of the population have intracranial aneurysms, and the rupture of aneurysm can lead to brain dysfunction or even death in patients.

**Methods:** To explore potential preventional target genes for the ruptured of aneurysm, we analyze three gene expression datasets (GSE13353, GSE15629 and GSE54083) derived from the GEO database. We confirm DEGs associated with the unrupture of aneurysms by R package. DAVID version provides functional classification and annotation analyses of associated genes, including GO and KEGG pathway. PPI of these DEGs is analyzed based on the string database and visualized by Cytoscape software. DEGs are verified by qRT-PCR using samples isolated from the patients.

**Results:** 249 overlapping DEGs, including 96 up-regulated genes and 153 down-regulated genes are screened using the Venn diagram webtool. The GO term and KEGG pathways analysis results indicate that these DEGs are mainly enriched in protein phosphorylation, apoptotic process and inflammatory response in the BP term and focal adhesion, thyroid hormone signaling pathway, ErbB signaling pathway, cytokine-cytokine receptor interaction and some disease processes in the KEGG pathways. 6 candidates are confirmed by Cytoscape software and qRT-PCR, including APP, JUN, GSK3B, ErbB2, PPBP and THBS1.

**Conclusions:** Our data and previous studies show that ErbB2 and THBS1 are crucial to prevent aneurysm rupture, while APP, JUN, GSK3B and PPBP performs the opposite role, and further experiments are needed to verify these findings.

## Background

Intracranial aneurysm (IA) is a saccular bulge in the wall of the artery, which is the first cause of spontaneous subarachnoid hemorrhage (SAH), accounting for 85% (Liu et al., 2019). Previous studies, using autopsy and angiography, had shown that 3.6–6% of the population had IAs. (Wardlaw and White, 2000; Macdonald and Schweizer, 2017; Qi et al., 2019). The estimate of rupture probability is vital to identifying at risk IA in patients. Many risk factors are associated with aneurysm rupture, including age, gender, hypertension, smoking, stress, geographic location, congenital vascular fragility, pathological conditions, inflammation and damage to the vessel wall (Liu et al., 2019). The rupture of aneurysm can lead to brain dysfunction or even death in patients, with a risk of 0.34% for patients with 1 aneurysm and 0.95% for those with several aneurysms (Liu et al., 2019). Therefore, It is desirable to find a suitable treatment for these patients. The first choice methods for the treatment of IAs are coil embolization and clipping. However, in the long term, these treatments can lead to serious consequences, such as the neurological complications, heavy economic burden and mental stress. Subsequently, it is crucial to understand the molecular mechanism of the rupture of IA. Pascale et al. demonstrated a link between oxidative stress in arterial walls and risk of rupture, and dimethyl fumarate significantly decreased the rupture rate of aneurysms (Pascale et al., 2020). Meanwhile, Kazuha and Yasuhisa et al. reported that Toll-like receptor 4 and the infiltration and polarization of immune-cell accelerated the rupture of IAs (Kanematsu et al., 2011; Mitsui et al., 2020). With the rapid development of gene chip technologies, Gene Expression Omnibus (GEO) are increasingly playing a essential role in bioinformatic analysis. In this study, we thereby sought to identify potential core genes and pathways of interest, which may ultimately provide potential therapeutic targets.

## Materials and Methods

### Acquisition of the Gene Expression Datasets

The gene expression datasets are obtained from the GEO database (https://www.ncbi.nlm.nih.gov/geo/). Three gene expression datasets (GSE13353, GSE15629 and GSE54083) of IAs are selected. Among them, GSE13353 is based on platform GPL570([HG-U133_Plus_2] Affymetrix Human Genome U133 Plus 2.0 Array), GSE15629 is based on platform GPL6244([HuGene-1_0-st] Affymetrix Human Gene 1.0 ST Array [transcript (gene) version]), and GSE54083 is based on platform GPL4133(Agilent-014850 Whole Human Genome Microarray 4 × 44 K G4112F (Feature Number version)). The data of GSE13353 dataset is submitted by Kurki MI et al., including 11 ruptured intracranial aneurysm samples (RIAs) and 8 unruptured intracranial aneurysm samples (UIAs) (Kurki et al., 2011). The data of GSE15629 dataset is submitted by Pera J et al., including 8 RIAs and 6 UIAs(Pera et al., 2010). The data of GSE54083 dataset is submitted by Nakaoka H et al., which consisted of 8 RIAs and 5 UIAs (Nakaoka et al., 2014)..(Table 1)

**TABLE 1:** Information for selected microarray datasets.

GEO Accession	Platform	Samples		Source Tissue
		UIAs	RIAs	
GSE13353	GPL570	8	11	Intracranial aneurysm sample
GSE15629	GPL6244	6	8	Intracranial aneurysm sample
GSE54083	GPL4133	5	8	Intracranial aneurysm sample

Annotation: GPL570 ([HG-U133_Plus_2] Affymetrix Human Genome U133 Plus 2.0 Array); GPL6244 ([HuGene-1_0-st] Affymetrix Human Gene 1.0 ST, Array [transcript (gene) version]); GPL4133 (Agilent-014850 Whole Human Genome Microarray 4 × 44 K G4112F (Feature Number version)).

### Identification of DEGs

The microarray data are processed and analyzed using the R package, which is used to detect the Differentially Expressed Genes (DEGs) between UIAs and RIAs. The median value of each sample is normalized using the limma package. Those genes with a *p*-value < 0.05 and absolute value of |log fold change (FC)| > 0.5 are considered for the DEGs. The overlapping of DEGs between datasets are obtained using an online Venn diagram webtool (http://bioinformatics.psb.ugent.be/webtools/Venn/). Statistical analysis is further evaluated for each dataset. The volcano plots and heatmap are visualized.

### GO and KEGG Pathway Analysis of DEGs

The database for Annotation, Visualization and Integrated Discovery (DAVID) version 6.8 (https://david.ncifcrf.gov/) provides functional classification and annotation analyses of associated genes, including GO and KEGG pathway (Huang et al., 2009). Gene Ontology (GO) analysis is a useful method for large scale functional enrichment research and GO analysis can be classified into biological process (BP), molecular function (MF), and cellular component (CC). Kyoto Encyclopedia of Genes and Genomes (KEGG) includes genomes, biological pathways, diseases, chemical, and metabolic information. A significance level of *p*-value < 0.05 is set as the cutoff criteria and the top five counts of each GOs and KEGGs are selected.

### PPI Network Construction and Module Analysis

The Search Tool for the Retrieval of Interacting Genes (STRING) version 11.5 database (http://string-db.org/) is designed to explore protein–protein interactions (PPI) (Szklarczyk et al., 2017). The DEGs identified previously are first mapped to the STRING database. Only the interactions with a combined score ≥0.4 are considered as significant. Subsequently, the PPI network is visualized using cytoscape 3.8.2 software and the hub nodes are identified by a high score based on the scale-free property of the network (Shannon et al., 2003). Furthermore, CytoHubba plugin provided a user-friendly interface to explore important nodes in biological networks, is utilized with the degree to explore the PPI network for hub genes. we performed quantitative expression analysis of 10 candidate genes which can better explain the results’ reliability. The Molecular Complex Detection (MCODE) plugin is used for searching the most significant module from the network. The MCODE criteria for selection are as follows: scores ≥4, degree cutoff = 2, node score cutoff = 0.2, K- core = 2, and max depth = 100.

### Patient Samples

A total of 6 tissue samples are obtained from the Department of Neurosurgery of Qingdao University Hospital and Changzhou Hospital of Tranditional Chinese Medicine. The research has been carried out in accordance with the World Medical Association Declaration of Helsinki. Simultaneously, it is approved by the Ethics Committee of Qingdao University Hospital and Changzhou Hospital of Tranditional Chinese Medicine, and informed consent is obtained from all of the recruited patients. All specimens are harvested and immediately frozen in liquid nitrogen and then stored at −80°C until the subsequent extraction of RNA.

### RNA Extraction and qRT-PCR

TRIzol reagent (Invitrogen, United States) is used to extract total RNA from the samples according to the manufacturer’s protocol. The concentration of purified RNA is examined using spectrophotometer (NanoDrop 2000, United States). Advantage^®^ RT-PCR Kit and random primers are used to synthesize cDNA (Clontech, Takara, Japan). Quantitative real-time PCR (qRT-PCR) is conducted on the LightCycler 480 Detection System with SYBR Green dye (Clontech, Takara, Japan). The PCR cycle conditions is as follows: a denaturation at 95°C for 30s; followed by 40 cycles of amplification (5 s at 95°C and 30 s at 60°C), finally at 65°C for15 s. Each sample is tested in triplicate, and each underwent a melting curve analysis to check the specificity of amplification (Cui et al., 2021). Supplementary Table S1 illustrates the primer sequences of hub genes.

### Statistical Analysis

GraphPad Prism software is employed to conduct the statistical test. All experiments are performed in triplicates. All data are presented as mean ± SD. A Student’s t-test is used to detect significant differences between groups. *p*-value < 0.05 is deemed as statistically significant.

## Results

### Identification of Ruptured IAs-Related DEGs

**FIGURE 1:**
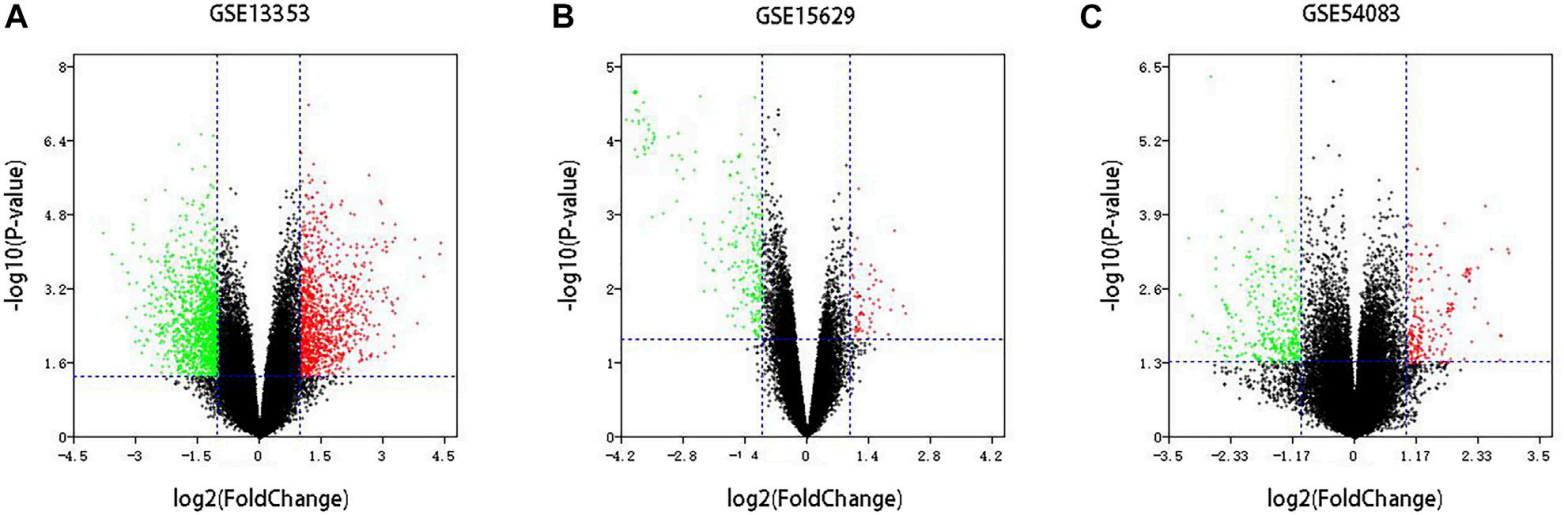
Volcano plot of DEGs among each GEO dataset. **(A–C)** Three GEO datasets.Red: up-regulated DEGs; Green: down-regulated DEGs; Black: unchanged genes.

**FIGURE 2:**
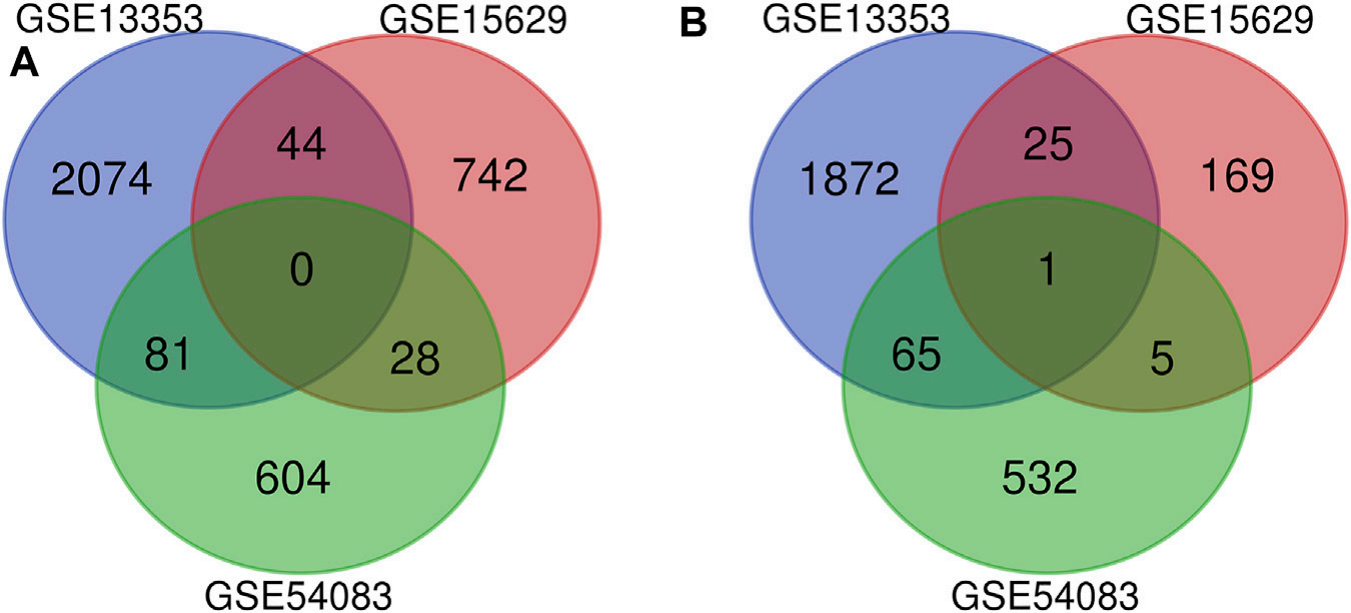
Venn diagrams of the DEGs in all three datasets. **(A)** 153 down-regulated DEGs screened out from the three datasets; **(B)** 96 up-regulated DEGs screened out from the three datasets.

Three datasets of ruptured IAs-related gene expression profiles (GSE15629, GSE13353 and GSE54083) are extracted from the GEO database. In order to integrate different experiments and sequencing platforms, the data need to be processed and standardized. A total of 1963, 200 and 603 up-regulated DEGs and 2199, 814 and 713 down-regulated DEGs are screened in GSE13353, GSE15629 and GSE54083 profiles by the R package (Figure 1). By using the Venn diagram webtool, we find 249 overlapping DEGs (≥2 datasets), including 96 up-regulated genes and 153 down-regulated genes (Figure 2). The Heatmap clearly illustrates the changes for these genes (Supplementary Figure S1).

### Functional and Pathway Enrichment Analyses of These DEGs

**FIGURE 3:**
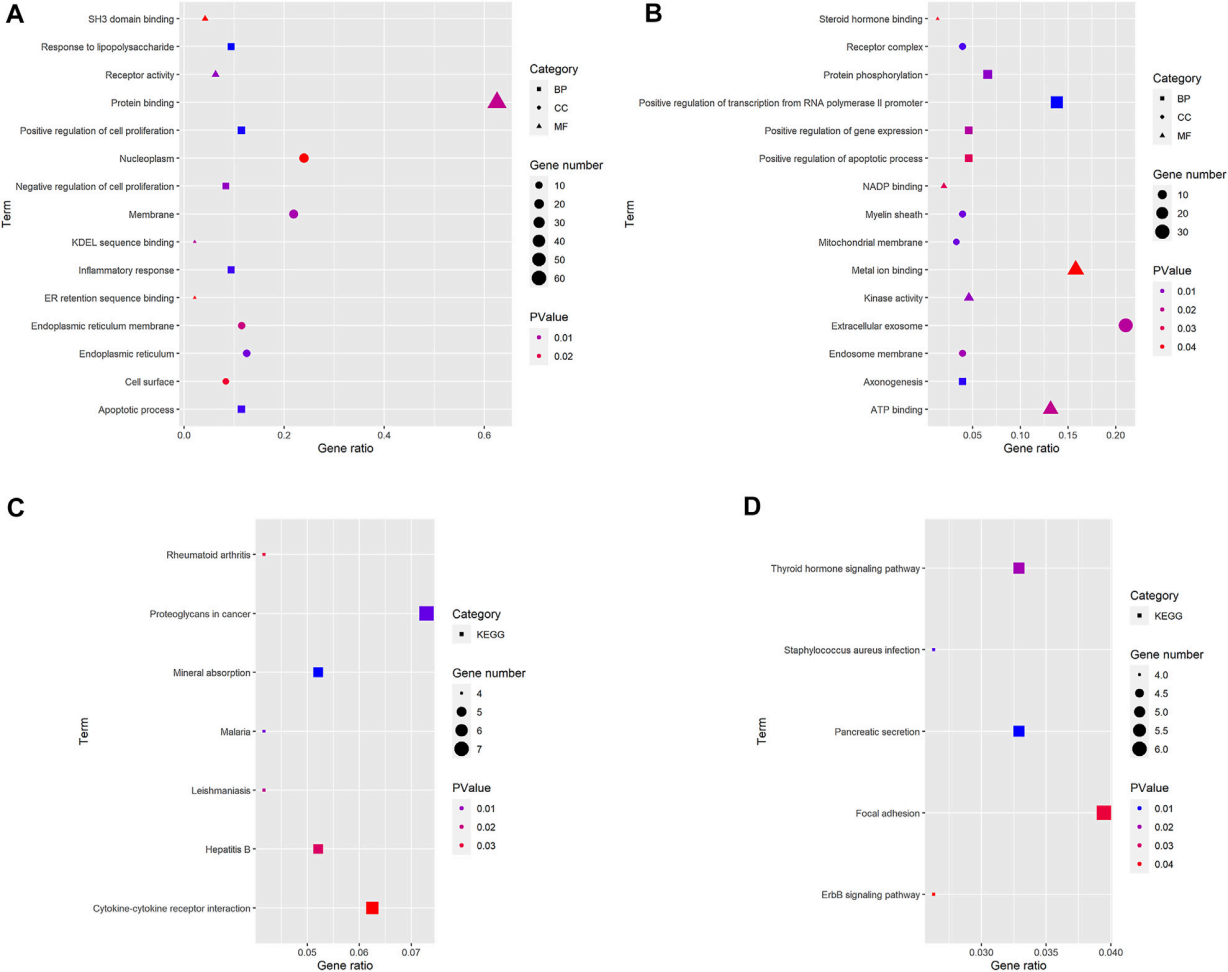
GO and KEGG pathway analyses results of DEGs. The *y*-axis shows GO terms or the KEGG pathways, and the gene number or *p*-value in the *x*-axis. **(A,C)** up-regulated DEGs; **(B,D)** down-regulated DEGs. BP: biological process; CC: cellular component; MF, molecular function; KEGG, Kyoto Encyclopedia of Genes and Genomes. *p*-value < 0.05.

**FIGURE 4:**
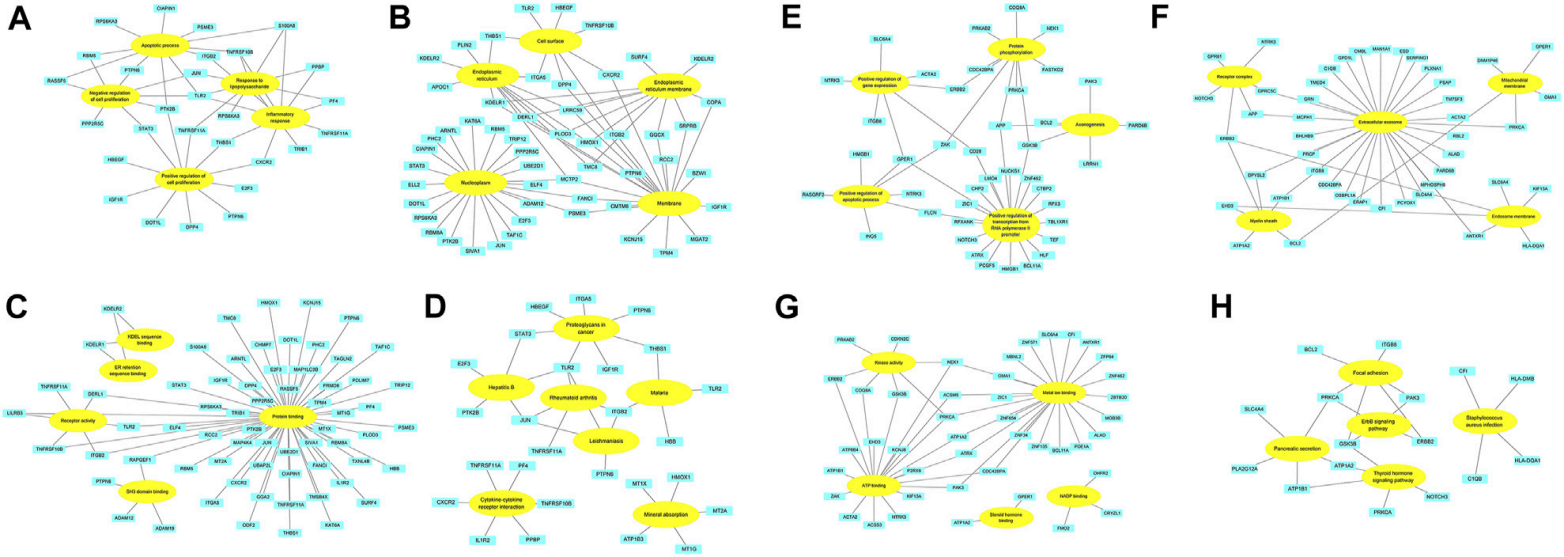
The association between David terms and DEGs. **(A–D)** BP, CC, MF and KEGG enriched by up-regulated DEGs; **(E–H)** BP, CC, MF and KEGG enriched by down-regulated DEGs. Yellow: GO or KEGG terms; Blue: DEGs.

Three terms of GO functional annotation analysis are performed on these 96 up-regulated and 153 down-regulated overlapping DEGs. With a threshold of *p*-value < 0.05, and top 5 significant GO and KEGG pathways show that the 96 up-regulated DEGs are enriched in 50 GO terms and 8 KEGG pathways. The enriched GO functions for these DEGs identify that the most significant terms are positive regulation of cell proliferation, apoptotic process, response to lipopolysaccharide, inflammatory response amd negative regulation of cell proliferation in the BP term; nucleoplasm, membrane, endoplasmic reticulum, endoplasmic reticulum membrane and cell surface in the CC term; protein binding, receptor activity, SH3 domain binding, KDEL sequence binding and ER retention sequence binding in the MF term. Subsequently, we conduct KEGG pathway enrichment analysis. The KEGG pathways display enrichment predominantly during poteoglycans in cancer, ctokine-cytokine receptor interaction, mneral absorption, hpatitis B, mlaria, lishmaniasis and reumatoid arthritis. (Supplementary Table S2). Meanwhile, the down-regulated DEGs are enriched in 29 GO terms and 9 KEGG pathways. The enriched GO functions for these DEGs identify the most significant terms are positive regulation of transcription from RNA polymerase II promoter, protein phosphorylation, positive regulation of gene expression, positive regulation of apoptotic process and axonogenesis in the BP term; extracellular exosome, receptor complex, myelin sheath, endosome membrane and mitochondrial membrane in the CC term; metal ion binding, ATP binding, kinase activity, NADP binding and steroid hormone binding in the MF term. In addition, the enriched KEGG pathways mainly contain focal adhesion, pancreatic secretion, thyroid hormone signaling pathway, *staphylococcus aureus* infection and ErbB (EGFR, epidermal growth factor receptor) signaling pathway. (Figures 3, 4, Supplementary Table S3).

### Construction and Analysis of PPI Network

**FIGURE 5:**
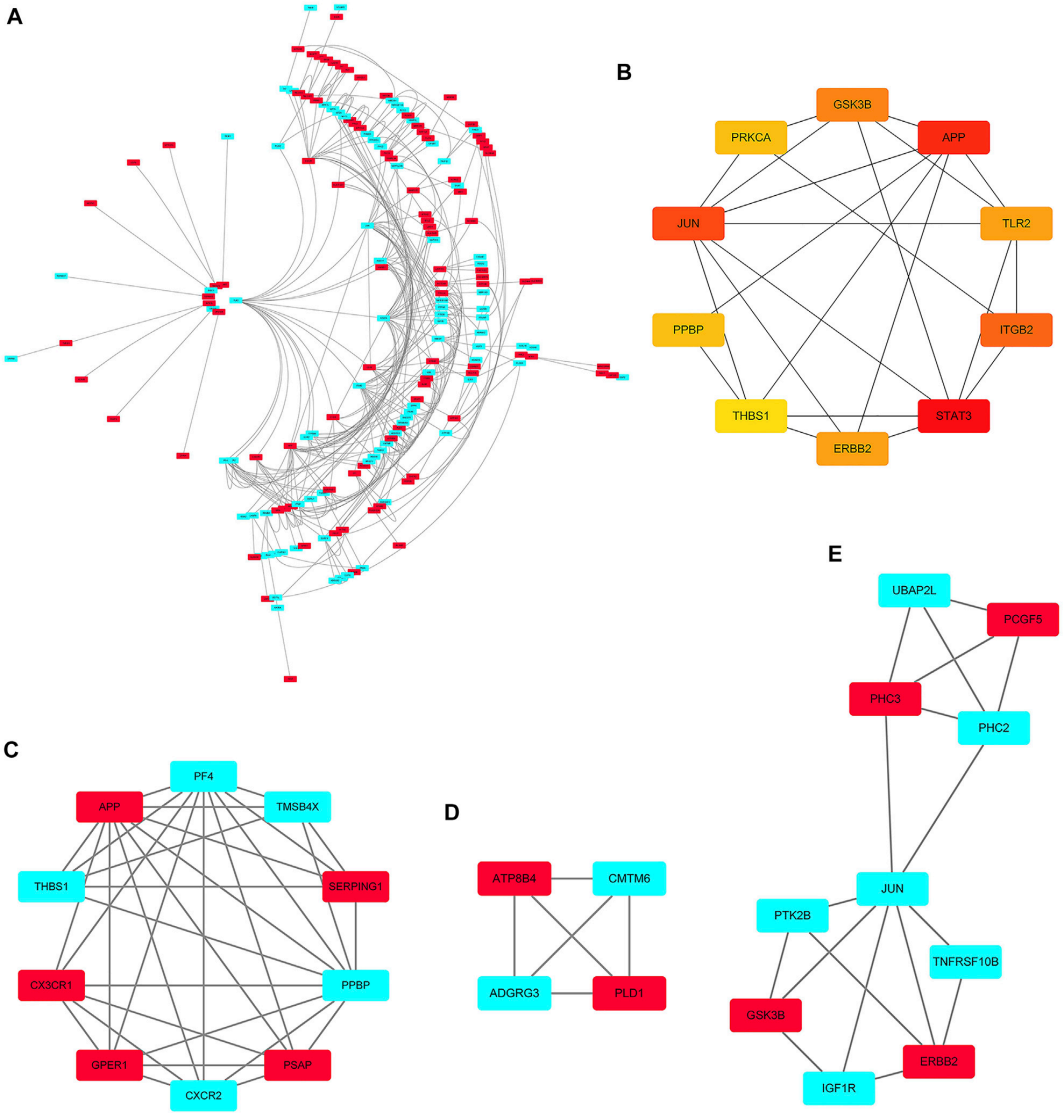
PPI network. **(A)** The PPI networks of 249 DEGs (153 down-regulated DEGs and 96 down-regulated DEGs); **(B)** The top 10 genes derived from the degree method are chosen using the CytoHubba plugin and the more forward ranking is represented by a redder color; **(C–E)** The top 3 significant modules obtained from the PPI network with a high score (≥4). Red: represent down-regulated DEGs; Blue: represent up-regulated DEGs.

The PPI networks of 249 DEGs are constructed using the STRING database. The subset of all DEGs (241 nodes and 359 edges) are included in the PPI network. In addition, those interactions are constructed by using cytoscape software (Figure 5A). Subsequently, we use MCODE plugin to explore meaningful modules in this network, including 3 modules in different subsets (Figures 5C–E; Table 2). The top 10 genes according to the degree are selected using the CytoHubba plugin and are ordered as follows: STAT3, APP, JUN, ITGB2, GSK3B, TLR2, ErbB2, PRKCA, PPBP and THBS1 (Figure 5B; Table 3). Notably, 6 of 10 above genes are included in the modules. Finally, we confirm 6 candidates for our further study: Thrombospondin1(THBS1), Amyloid precursor protein (APP), Pro–platelet basic protein (PPBP), Erb-B2 receptor tyrosine kinase 2 (ErbB2), Glycogen synthase kinase 3 beta (GSK3B) and Jun proto-oncogene (JUN).

**TABLE 2:** Modules associated DEGs extracted by MCODE.

Cluster	Score (Density*#Nodes)	Nodes	Edges	Gene Symbol
1	7.333	10	33	THBS1, CX3CR1, CXCR2, PSAP, APP, TMSB4X, PF4, SERPING1, GPER1, PPBP
2	4	4	6	ADGRG3, CMTM6, ATP8B4, PLD1
3	4	10	18	ERBB2, IGF1R, GSK3B, JUN, UBAP2L, PHC2, PHC3, PCGF5, PTK2B, TNFRSF10B

**TABLE 3:** Top 10 in network ranked by Degree method.

Rank	Name	Degree
1	STAT3	24
2	APP	22
3	JUN	21
4	ITGB2	20
5	GSK3B	17
6	TLR2	16
7	ERBB2	16
8	PRKCA	13
9	PPBP	13
10	THBS1	12

### The mRNA Levels of Hub Genes

**FIGURE 6:**
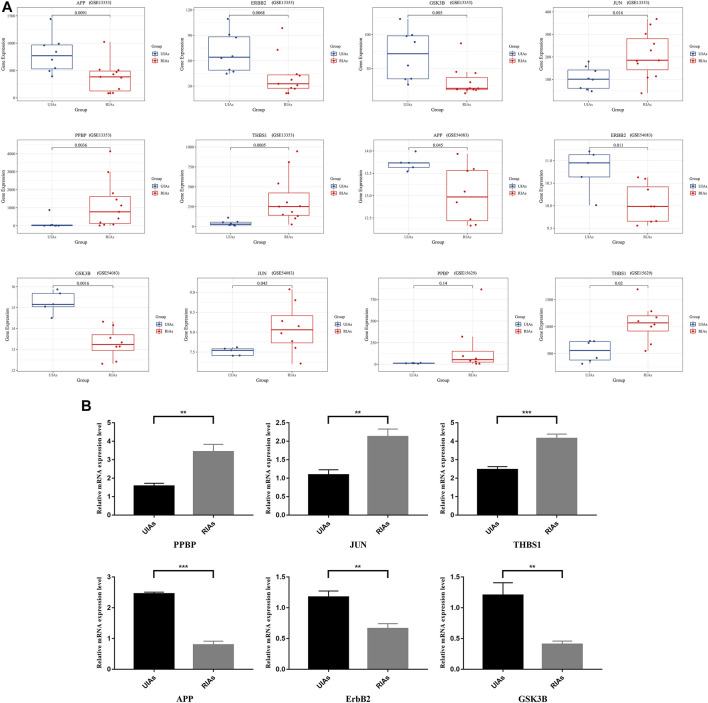
Relative expression level of six hub genes in UIAs and RIAs. **(A)** The expression level of hub genes in GEO dataset; **(B)** The mRNA levels of hub genes detected by qRT-PCR. The *y*-axis represents the relative expression of genes. UIAs: Unruptured Intracranial Aneurysm Samples; RIAs: Ruptured Intracranial Aneurysm Samples. ∗*p*-value < 0.05, ∗∗*p* < 0.01, ∗∗∗*p* < 0.001.

**FIGURE 7:**
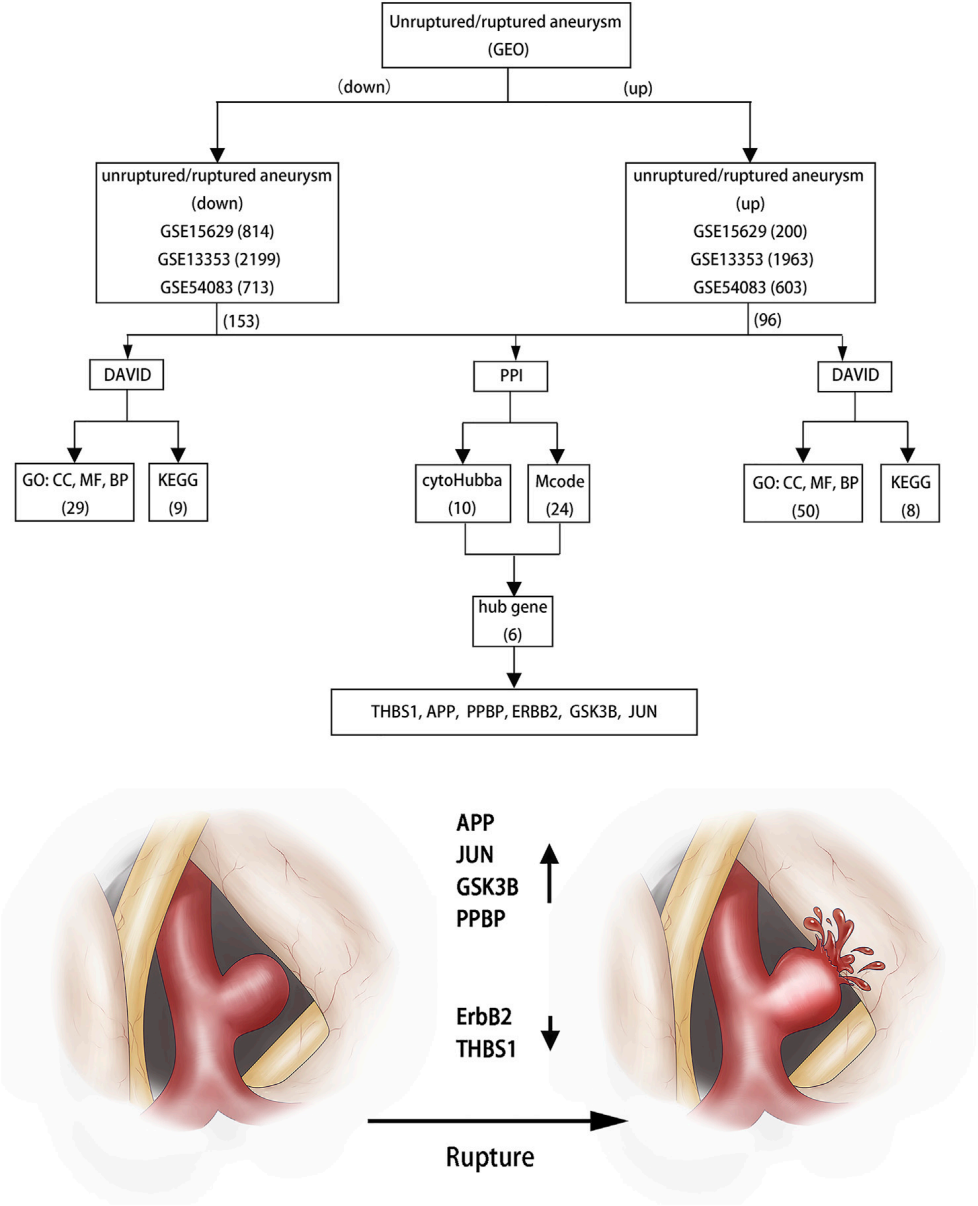
Flowchart of bioinformatics analysis.

Through the visualized data, we can clearly observe the expression of APP, JUN, GSK3B, ErbB2, PPBP and THBS1. Figure 6A illustrates the expression of statistically significant hub genes. To further validate the hub genes, the mRNA levels of these hub genes (APP, JUN, GSK3B,ErbB2, PPBP and THBS1) in 6 samples are determined, and the results are consistent with previous findings. As illustrated in Figure 6B, the level of THBS1, PPBP, and JUN expression is elevated and the expression of APP, GSK3B and ErbB2 is significantly lower in patients compared with controls. These results confirm that six hub genes are potential biomarker for ruptured aneurysms (Figure 7).

## Discussion

The rupture of aneurysm could lead to brain dysfunction or eventual mortality, with some potential risks (Liu et al., 2019). Although nonsurgical treatment for IAs is much awaited, few schemes are approachable because its molecular pathogenesis remains elusive. Many factors affect the rupture of IAs by altering morphologies in the cerebral vasculature. Understanding the molecular mechanism of the rupture of IAs may facilitate the development of novel therapeutic strategy.

Thus, we identify 249 overlapping DEGs, which consist of 96 up-regulated genes and 153 down-regulated genes. Moreover, the GO analysis suggest that these up-regulated and down-regulated genes are mostly involved in protein phosphorylation, apoptotic process and inflammatory response. Furthermore, KEGG pathway enrichment analysis show that these DEGs are significantly enriched in focal adhesion, thyroid hormone signaling pathway, ErbB signaling pathway and cytokine-cytokine receptor interaction. Xu et al. identified that cell adhesion indicated a more universal validity in IAs (Xu et al., 2019). Similarly, apoptosis, as a programmed process of cell death, play an important role in many aspects. And, previous studies showed that vascular endothelial cells apoptosis contributed to vessel pruning and the elasticity of vessel was impaired along with multiple apoptosis of vascular smooth muscle cells (Zhang et al., 2018; Xue et al., 2019). At the same time, we find that these DEGs are enriched in some inflammatory reactions. And Signorelli et al. had proved that inflammation were also a key factor leading to the formation and rupture of aneurysm (Signorelli et al., 2018). Alicia et al. suggested that ErbB2/neu might stimulate angiogenesis by up-regulating potent angiogenesis growth factors such as vascular endothelial growth factor/vascular permeability factor (VEGF/VPF) (Petit et al., 1997). Enrichment analysis show that most of these DEGs contribute to a structural disorder in the blood vessels, which leading to the rupture of aneurysms.

Subsequently, 6 hub genes (APP, JUN, GSK3B, ErbB2, PPBP and THBS1) are selected by the clustering analysis. The Heatmap and Box figure clearly exhibits that APP, GSK3B and ErbB2 are down-regulated and THBS1, PPBP, and JUN are up-regulated in these profiles.

APP is central to the amyloid cascade hypothesis wherein APP is cleaved by β- and γ-secretases to form plaques and amyloid-β (Aβ) peptides, leading to Alzheimer’s disease (Selkoe and Hardy, 2016; Lee et al., 2018). After multivariable adjustment, evidence showed that the presence of plaque was associated with 1.31 times higher risk of abdominal aortic aneurysm (AAA) (Yao et al., 2018). Moreover, invaluable research showed that APP gene deposited and produced dysfunction in cerebral vessels, which lead to endothelial cell death (Price et al., 1997). Van et al. identified this kind of change culminated with weakening of the vessel all and aneurysm formation (Van Dorpe et al., 2000). What’s more, blood leakage in the vessel wall (Pfeifer et al., 2002; van Veluw et al., 2021) and various effective immune factors (Welikovitch et al., 2020) had been reported preciously in some APP transgenic models. All of this will eventually lead to increased fragility of the blood vessel wall.

PPBP and its many derivatives are potent neutrophil chemoattractants and activators. PPBP plays a significant role in atherosclerotic plaque formation (Apostolakis and Spandidos, 2013; Perisic et al., 2016), which causes the vessels to become more vulnerable to external forces at this site.

GSK3b is a vital downstream signaling member of glutamate insult, which can be activated at two phosphorylation sites (Tyr216 and Ser9) (Beurel et al., 2015). It was reported that GSK3b represented a switch of mitochondrial fuel influx and a potential target for anti-inflammatory therapy (Zeisbrich et al., 2018). Furthermore, the current scientific data identifies inactivation of GSK3b as central regulator of metabolic reprogramming in macrophages infiltrating into the atherosclerotic plaque (Zeisbrich et al., 2018). GSK3b may play a negative role in the rupture pathogenesis of aneurysms.

Thbs1 is a critical component of mechanotransduction as well as a modulator of elastic fiber organization, and which is a homotrimeric glycoprotein secreted by various cells, including vascular smooth muscle cells (SMCs) and endothelial cells (ECs) (Yamashiro et al., 2018). Thbs1 is maintained at low levels in the postnatal vessels and elevated in various vascular diseases, including pulmonary arterial hypertension (Kumar et al., 2017), aortic aneurysms (Kessler et al., 2014), atherosclerosis (Moura et al., 2008), cerebral cavernous malformations (Lopez-Ramirez et al., 2017) and ischemia reperfusion injury (Favier et al., 2005). Thbs1 negatively regulates cell adhesion, migration, proliferation, cell-cell interaction and angiogenesis, while positively regulates inflammation and activation of latent TGFβ. This broad spectrum of biological functions is attributable to domain structures with distinct binding sites for various extracellular matrix (ECM) molecules and cell surface receptors, such as integrins, CD36 and CD47 (Adams and Lawler, 2011; Resovi et al., 2014; Soto-Pantoja et al., 2015). Recent investigations strongly suggest that Thbs1 contributes to the development of abdominal aortic aneurysm (AAA) through acceleration of vascular inflammation (Yamashiro et al., 2018). Maladaptive upregulation of Thbs1 results in disruption of elastin-contractile units and dysregulation of actin cytoskeletal remodeling, contributing to the development of ascending aortic aneurysms *in vivo* (Yamashiro et al., 2018). Thus, deletion of Thbs1 suppresses abnormal mechanotransduction in the aneurysmal wall.

C-Jun is a proto-oncogene that participates in various cellular processes, including cell proliferation, apoptosis and differentiation (Reiner et al., 2011), which is required for cell cycle progression through the G1 phase (Wisdom et al., 1999). C-Jun N-terminal kinase (JNK) is a protein kinase that promotes the phosphorylation sites (Ser63 and Ser73) in the active region of the c-Jun amino terminal. Yoshimura et al. identified that JNK as a premier signaling molecule in the pathogenesis of aneurysm by suppressing the synthesis of biological enzymes while enhancing the degeneration of the extracellular matrix (Yoshimura et al., 2005; Yoshimura et al., 2006). Similarly, the activation of JNK also induces the production of pro-inflammatory signaling molecule (Chen et al., 2020; Kurashiki et al., 2020).

ErbB2 as a member of the ErbB family of transmembrane receptor tyrosine kinases, can be activated by forming heterodimers with ligand-occupied ErbBs, leading to increased kinase activity and downstream signaling molecule transduction (Sinha et al., 2003). ErbB2 is pivotal regulators of cell migration, differentiation, proliferation and survival. Zhang et al. indicated that ErbB2 play an vital cytoprotective roles in the colonic epithelium by protecting against epithelial apoptosis and reducing colonic expression of tumor necrosis factor alpha (TNF-α) (Zhang et al., 2012). Furthermore, Zhang et al. suggested that ErbBs-mediated signaling transduction play a critical role in maintaining the integrality of blood vessel endothelium and mesenteric vascular dysfunction (Zhang et al., 2019).

In essence, prior studies have noted that the rupture of aneurysms is mainly related to inflammatory factors, plaque and apoptosis. These factors can induce the fragility of the aneurysm wall or the formation of the vessel gap, and ultimately induce high risk of the aneurysm rupture. If we can enhance the elasticity of blood vessel walls through these signaling molecules, the risk of aneurysm rupture and surgery complications will be reduced. Based on the above analysis, we can infer that APP, JUN, GSK3B and PPBP can induce the rupture of aneurysms, while ErbB2 and THBS1 will play a protective role. Important to note here is that these hub genes play various roles in the rupture of aneurysms and these result provide detailed clues for understanding the molecular mechanism of the pathogenesis of aneurysms and identify potential therapeutic targets. However, the real role of these genes in aneurysms is not clear and molecular biological experiments are needed to verify these findings.

## Conclusion

This bioinformatics analysis identified DEGs obtained from the GEO databases. Among them, six hub genes might be the core genes for the rupture of aneurysms, including APP, JUN, GSK3B,ErbB2, PPBP and THBS1. As a consequence we infer that ErbB2 and THBS1 are crucial to prevent aneurysm rupture, while APP, JUN, GSK3B and PPBP performs the opposite role. These six genes provide new potential therapeutic targets for preventing aneurysm rupture.

## Data Availability

The original contributions presented in the study are included in the article/Supplementary Material, further inquiries can be directed to the corresponding author.

## Abbreviations

BP, Biological Process; CC, Cellular Component; DEGs, Differentially Expressed Genes; ErbB2, Erb-B2 receptor tyrosine kinase 2; GEO, Gene Expression Omnibus; GO, Gene Ontology; GSK3B, Glycogen synthase kinase 3 beta; IAs, Intracranial Aneurysms; JUN, Jun proto-oncogene, KEGG, Kyoto Encyclopedia of Genes and Genomes, MCC, Maximal Clique Centrality; MCODE, Molecular Complex Detection; MF; Molecular Function, PPBP, Pro–platelet Basic Protein; RIAs, Ruptured Intracranial Aneurysm Samples, SAH; Subarachnoid Hemorrhage, THBS1, Thrombospondin1; UIAs, Unruptured Intracranial Aneurysm Samples; VEGF, Vascular Endothelial Growth Factor; VPF, Vascular Permeability Factor.
