# Supplementary material for: Identification of Potential Core Genes for the Rupture of Intracranial Aneurysms by a Bioinformatics Analysis
Source: Front Genet. 2022 Mar 30;13:875007. doi: 10.3389/fgene.2022.875007 (PMC9006073; doi:10.3389/fgene.2022.875007)
Supplement: Supplementary file 1 [file Table1.DOCX]

**S1. The primer sequences information**

| Gene | Forward (5′‐3′) | Reverse (5′‐3′) |
| --- | --- | --- |
| ERBB2 | CCAGCCTTCGACAACCTCTATT | TGCCGTAGGTGTCCCTTTG |
| PPBP | TTGTAGGCAGCAACTCACCC | TGCAAGGCATGAAGTGGTCT |
| Thbs1 | ACCGGTTATATCAGAGTGGTGATG | TGTCTGAGAAGAACACCATTTCCT |
| APP | TCTCGTTCCTGACAAGTGCAA | GCAAGTTGGTACTCTTCTCACTG |
| GSK-3 | ATTTTCCAGGGGATAGTGGTGT | GGTCGGAAGACCTTAGTCCAAG |
| JUN | CTGTTCTATGACTGCAAAGATG | GGGTTACTGTAGCCATAAGGT |
| β-actin | CCTCGCCTTTGCCGATCC | GGATCTTCATGAGGTAGTCAGTC |
