# Supplementary figures and images for: Identification of Potential Core Genes for the Rupture of Intracranial Aneurysms by a Bioinformatics Analysis
Source: Front Genet. 2022 Mar 30;13:875007. doi: 10.3389/fgene.2022.875007 (PMC9006073; doi:10.3389/fgene.2022.875007)

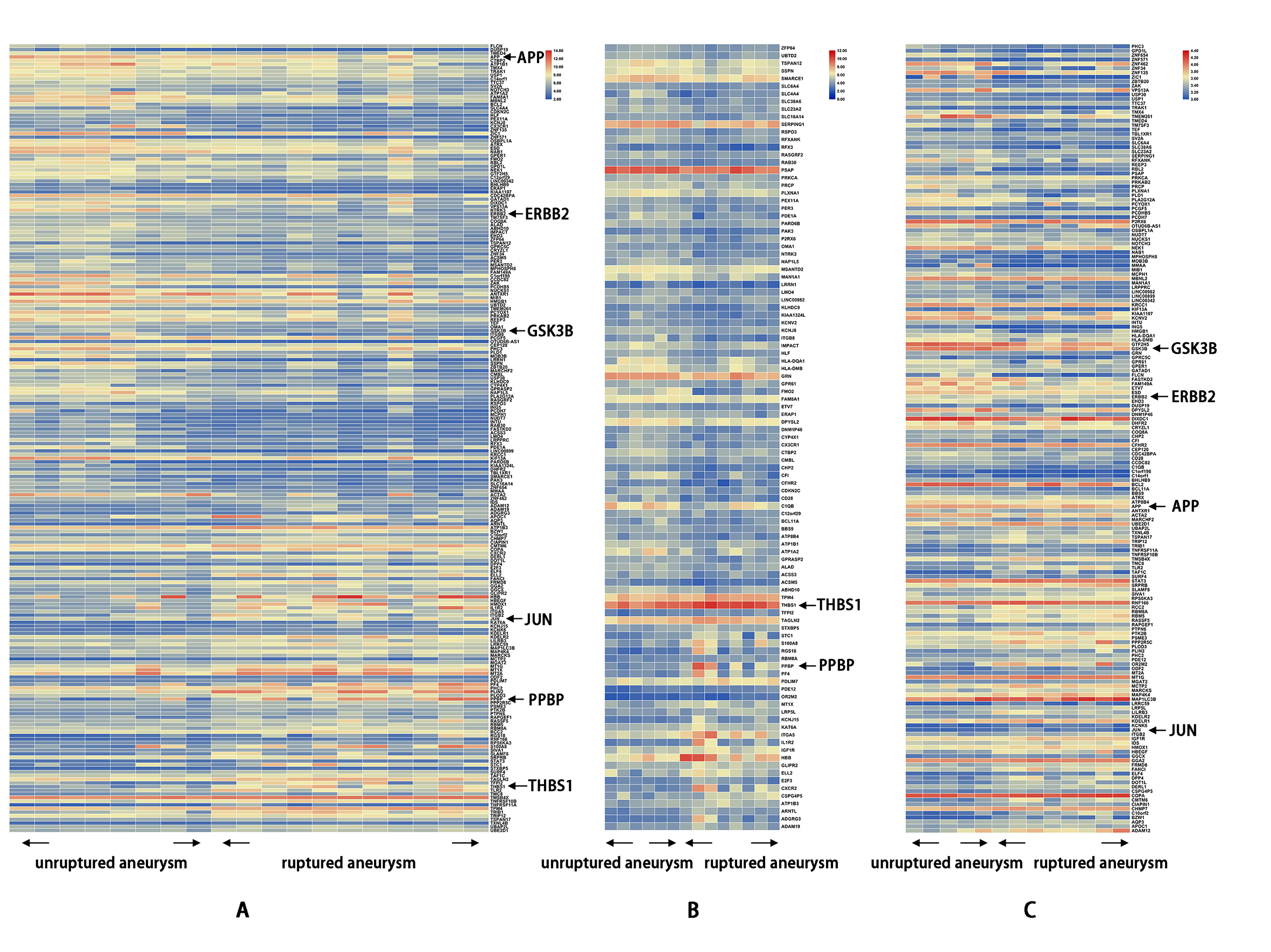

Supplement: Supplementary file 2 [file Image1.TIF]
