# Supplementary material for: Identification of Potential Core Genes for the Rupture of Intracranial Aneurysms by a Bioinformatics Analysis
Source: Front Genet. 2022 Mar 30;13:875007. doi: 10.3389/fgene.2022.875007 (PMC9006073; doi:10.3389/fgene.2022.875007)
Supplement: Supplementary file 3 [file Table2.DOCX]

**S2. GO Terms and KEGG Pathway in up-regulated DEGs**

| Category | Term | Count | PValue | Genes |
| --- | --- | --- | --- | --- |
| BP | Positive regulation of cell proliferation | 11 | 0.000253689 | DOT1L, IGF1R, PTPN6, E2F3, TNFRSF11A, PTK2B, HBEGF, CXCR2, THBS1, DPP4, STAT3 |
| BP | Apoptotic process | 11 | 0.001162444 | PTPN6, RASSF5, RPS6KA3, TNFRSF10B, S100A8, PTK2B, TLR2, RBM5, PSME3, ITGB2, CIAPIN1 |
| BP | Response to lipopolysaccharide | 9 | 3.51347E-06 | RPS6KA3, TNFRSF11A, TNFRSF10B, S100A8, PPBP, JUN, TLR2, PF4, TRIB1 |
| BP | Inflammatory response | 9 | 0.001199584 | TNFRSF11A, TNFRSF10B, S100A8, PPBP, TLR2, CXCR2, ITGB2, PF4, THBS1 |
| BP | Negative regulation of cell proliferation | 8 | 0.006414807 | PTPN6, RASSF5, PTK2B, JUN, PPP2R5C, TLR2, RBM5, STAT3 |
| CC | Nucleoplasm | 23 | 0.027466918 | SIVA1, TAF1C, E2F3, ELF4, PPP2R5C, RBM5, ARNTL, CIAPIN1, STAT3, ELL2, DOT1L, RPS6KA3, MCTP2, FANCI, RBM8A, PTK2B, JUN, PSME3, ADAM12, UBE2D1, TRIP12, KAT6A, PHC2 |
| CC | Membrane | 21 | 0.008805586 | GGCX, COPA, PTPN6, KCNJ15, DERL1, CXCR2, ITGB2, SRPRB, TPM4, MGAT2, IGF1R, BZW1, MCTP2, RCC2, FANCI, HMOX1, LRRC59, PSME3, KDELR1, DPP4, CMTM6 |
| CC | Endoplasmic reticulum | 12 | 0.003838165 | KDELR2, DERL1, MCTP2, TMC8, PLIN2, ITGA5, PLOD3, HMOX1, LRRC59, APOC1, THBS1, KDELR1 |
| CC | Endoplasmic reticulum membrane | 11 | 0.014276782 | GGCX, COPA, KDELR2, DERL1, TMC8, PLOD3, HMOX1, LRRC59, SURF4, SRPRB, KDELR1 |
| CC | Cell surface | 8 | 0.023148695 | TNFRSF10B, ITGA5, TLR2, HBEGF, CXCR2, ITGB2, THBS1, DPP4 |
| MF | Protein binding | 60 | 0.012655734 | TAF1C, KCNJ15, E2F3, PDLIM7, S100A8, ELF4, CHMP7, PPP2R5C, RBM5, TLR2, CXCR2, CIAPIN1, TNFRSF11A, FANCI, RBM8A, FRMD8, MAP1LC3B, HMOX1, PLOD3, RAPGEF1, DPP4, PHC2, ARNTL, MAP4K4, TNFRSF10B, RCC2, JUN, LILRB3, SURF4, TMSB4X, PSME3, SIVA1, IL1R2, DERL1, PF4, ITGB2, TAGLN2, TPM4, TRIB1, IGF1R, PTK2B, UBAP2L, ODF2, THBS1, UBE2D1, HBB, MT1G, TRIP12, PTPN6, TXNL4B, TMC8, MT1X, STAT3, DOT1L, RASSF5, RPS6KA3, ITGA5, MT2A, GGA2, KAT6A |
| MF | Receptor activity | 6 | 0.006671716 | TNFRSF11A, DERL1, TNFRSF10B, LILRB3, TLR2, ITGB2 |
| MF | SH3 domain binding | 4 | 0.027252938 | PTPN6, ADAM19, ADAM12, RAPGEF1 |
| MF | KDEL sequence binding | 2 | 0.010870448 | KDELR2, KDELR1 |
| MF | ER retention sequence binding | 2 | 0.026957328 | KDELR2, KDELR1 |
| KEGG | Proteoglycans in cancer | 7 | 0.004122283 | IGF1R, PTPN6, ITGA5, TLR2, HBEGF, THBS1, STAT3 |
| KEGG | Cytokine-cytokine receptor interaction | 6 | 0.038232091 | IL1R2, TNFRSF11A, TNFRSF10B, PPBP, CXCR2, PF4 |
| KEGG | Mineral absorption | 5 | 0.000339426 | ATP1B3, HMOX1, MT2A, MT1X, MT1G |
| KEGG | Hepatitis B | 5 | 0.024973443 | E2F3, PTK2B, JUN, TLR2, STAT3 |
| KEGG | Malaria | 4 | 0.006197746 | TLR2, ITGB2, THBS1, HBB |
| KEGG | Leishmaniasis | 4 | 0.017078324 | PTPN6, JUN, TLR2, ITGB2 |
| KEGG | Rheumatoid arthritis | 4 | 0.029936892 | TNFRSF11A, JUN, TLR2, ITGB2 |
