# Supplementary material for: Identification of Potential Core Genes for the Rupture of Intracranial Aneurysms by a Bioinformatics Analysis
Source: Front Genet. 2022 Mar 30;13:875007. doi: 10.3389/fgene.2022.875007 (PMC9006073; doi:10.3389/fgene.2022.875007)
Supplement: Supplementary file 4 [file Table3.DOCX]

**S3. GO Terms and KEGG Pathway in down-regulated DEGs**

| Category | Term | Count | PValue | Genes |
| --- | --- | --- | --- | --- |
| BP | Positive regulation of transcription from RNA polymerase II promoter | 21 | 7.21E-05 | HMGB1, HLF, TBL1XR1, CTBP2, NUCKS1, LMO4, CHP2, GPER1, ZIC1, FLCN, RFXANK, NOTCH3, ATRX, PCGF5, APP, GSK3B, BCL11A, TEF, RFX3, ZNF462, CD28 |
| BP | Protein phosphorylation | 10 | 0.009508 | PRKCA, APP, FASTKD2, ZAK, GSK3B, NEK1, ERBB2, COQ8A, PRKAB2, CDC42BPA |
| BP | Positive regulation of gene expression | 7 | 0.016886 | NTRK3, ITGB8, ACTA2, ERBB2, SLC6A4, GPER1, CD28 |
| BP | Positive regulation of apoptotic process | 7 | 0.030284 | ING5, NTRK3, HMGB1, ZAK, RASGRF2, GPER1, FLCN |
| BP | Axonogenesis | 6 | 0.001018 | PARD6B, APP, PAK3, BCL2, GSK3B, LRRN1 |
| CC | Extracellular exosome | 32 | 0.017996 | ATP1B1, ALAD, GPRC5C, TM7SF3, PLXNA1, ESD, CMBL, GPD1L, TMED4, APP, BHLHB9, ITGB8, OSBPL1A, ERAP1, CFI, SLC4A4, PRKCA, PARD6B, RBL2, ACTA2, PSAP, SERPING1, MAN1A1, DPYSL2, C1QB, GRN, MCPH1, PRCP, CDC42BPA, ANTXR1, PCYOX1, MPHOSPH8 |
| CC | Receptor complex | 6 | 0.002813 | NOTCH3, NTRK3, APP, GPRC5C, GPR61, ERBB2 |
| CC | Myelin sheath | 6 | 0.006032 | ATP1B1, BCL2, ERBB2, DPYSL2, ATP1A2, EHD3 |
| CC | Endosome membrane | 6 | 0.013389 | KIF13A, ERBB2, SLC6A4, ANTXR1, EHD3, HLA-DQA1 |
| CC | Mitochondrial membrane | 5 | 0.005673 | DNM1P46, PRKCA, BCL2, GPER1, OMA1 |
| MF | Metal ion binding | 24 | 0.044025 | PRKCA, ALAD, ZBTB20, ZFP64, NEK1, SLC6A4, MBNL2, ATP1A2, ZIC1, ZNF654, ZNF34, ATRX, PAK3, ZNF135, BCL11A, PDE1A, CDC42BPA, MOB3B, ZNF462, ANTXR1, CFI, ZNF571, ACSM5, OMA1 |
| MF | ATP binding | 20 | 0.020394 | PRKCA, ATP1B1, ZAK, ACTA2, ERBB2, NEK1, ATP1A2, ACSS3, NTRK3, KIF13A, ATRX, P2RX6, PAK3, KCNJ8, GSK3B, COQ8A, CDC42BPA, ACSM5, EHD3, ATP8B4 |
| MF | Kinase activity | 7 | 0.010934 | PRKCA, CDKN2C, GSK3B, NEK1, ERBB2, COQ8A, PRKAB2 |
| MF | NADP binding | 3 | 0.029671 | FMO2, DHFR2, CRYZL1 |
| MF | Steroid hormone binding | 2 | 0.037921 | ATP1A2, GPER1 |
| KEGG | Focal adhesion | 6 | 0.037406 | PRKCA, PAK3, ITGB8, BCL2, GSK3B, ERBB2 |
| KEGG | Pancreatic secretion | 5 | 0.009609 | PRKCA, ATP1B1, PLA2G12A, ATP1A2, SLC4A4 |
| KEGG | Thyroid hormone signaling pathway | 5 | 0.019658 | NOTCH3, PRKCA, ATP1B1, GSK3B, ATP1A2 |
| KEGG | Staphylococcus aureus infection | 4 | 0.012479 | C1QB, CFI, HLA-DMB, HLA-DQA1 |
| KEGG | ErbB signaling pathway | 4 | 0.043308 | PRKCA, PAK3, GSK3B, ERBB2 |
